# Senegenin Attenuates Pulmonary Fibrosis by Inhibiting Oxidative-Stress-Induced Epithelial Cell Senescence through Activation of the Sirt1/Pgc-1α Signaling Pathway

**DOI:** 10.3390/antiox13060675

**Published:** 2024-05-31

**Authors:** Qian Zeng, Yuyang Luo, Xiaoxue Sang, Minlin Liao, Binbin Wen, Zhengang Hu, Mei Sun, Ziqiang Luo, Xiaoting Huang, Wei Liu, Siyuan Tang

**Affiliations:** 1 Xiangya Nursing School, Central South University, Changsha 410013, China; zengqian02@csu.edu.cn (Q.Z.); 227811043@csu.edu.cn (Y.L.); sangsang@csu.edu.cn (X.S.); liaominlin120@csu.edu.cn (M.L.); binbin.wen@csu.edu.cn (B.W.); sunmei@csu.edu.cn (M.S.); huangxiaoting@csu.edu.cn (X.H.); 2 Xiangya School of Medicine, Central South University, Changsha 410013, China; huhuhu@csu.edu.cn (Z.H.); luoziqiang@csu.edu.cn (Z.L.)

**Keywords:** senegenin, pulmonary fibrosis, epithelial cell senescence

## Abstract

Idiopathic pulmonary fibrosis is a fatal interstitial lung disease for which effective drug therapies are lacking. Senegenin, an effective active compound from the traditional Chinese herb *Polygala tenuifolia Willd*, has been shown to have a wide range of pharmacological effects. In this study, we investigated the therapeutic effects of senegenin on pulmonary fibrosis and their associated mechanisms of action. We found that senegenin inhibited the senescence of epithelial cells and thus exerted anti-pulmonary-fibrosis effects by inhibiting oxidative stress. In addition, we found that senegenin promoted the expression of Sirt1 and Pgc-1α and that the antioxidative and antisenescent effects of senegenin were suppressed by specific silencing of the *Sirt1* and *Pgc*–*1α* genes, respectively. Moreover, the senegenin-induced effects of antioxidation, antisenescence of epithelial cells, and antifibrosis were inhibited by treatment with Sirt1 inhibitors in vivo. Thus, the Sirt1/Pgc-1α pathway exerts its antifibrotic effect on lung fibrosis by mediating the antioxidative and antisenescent effects of senegenin.

## 1. Introduction

Idiopathic pulmonary fibrosis (IPF) is a chronic progressive interstitial lung disease, and its incidence and prevalence are increasing globally with an aging population [1,2]. Currently, there is a lack of specific drugs that can effectively treat IPF. Nintedanib and pirfenidone, the only two medicines approved by the Food and Drug Administration for the clinical treatment of IPF, cannot reverse pulmonary fibrosis and have multiple side effects [3,4]. Therefore, research on the pathogenesis of IPF and the development of specific medicines for its treatment are of great significance.

Cellular senescence is usually defined as irreversible cell cycle arrest associated with changes in cellular morphology, secretory profiles, and epigenetic alterations [5,6]. Senescent cells experience irreversible replication arrest, apoptosis resistance, increased reactive oxygen species production, and increased abundance of senescence-associated secretory phenotypes, all of which affect normal cellular physiological functions [7]. Cellular senescence has been shown to be a key pathogenic mechanism in a variety of chronic diseases, and inhibition of cellular senescence can effectively inhibit the progression of these diseases [8]. In particular, epithelial cell senescence has been a research hot spot in recent years, and inhibition of epithelial cell senescence has also been shown to be effective in treating the progression of multiple chronic respiratory and urologic diseases [9,10,11,12].

Although the etiology and pathogenesis of IPF are currently unknown, an increasing number of studies have shown that alveolar epithelial cell senescence promotes the development of IPF, whereas its inhibition effectively suppresses the progression of pulmonary fibrosis. This may be related to the fact that the key secretory and regenerative roles of senescent alveolar epithelial cells in the alveoli are compromised, leading to impairment of their role in maintaining lung homeostasis [13]. Multiple antiaging mechanisms in the body play an important role in inhibiting the progression of IPF due to the senescence of epithelial cells. Among them, Sirtuin proteins including Sirt1, Sirt3, and Sirt6 have been shown to play key roles; however, their detailed mechanisms have yet to be explored [14,15,16].

Senegenin, a major bioactive component isolated from the roots of *Polygala tenuifolia Willd* [17], has been shown to have a wide range of pharmacological effects, including attenuating acute lung injury, improving cognitive impairment, and promoting the repair of spinal cord injury [18,19,20]. However, its use in the treatment of pulmonary fibrosis has not been studied. In the present study, we investigated for the first time the anti-pulmonary-fibrosis effect of senegenin and elucidated the mechanism of its anti-pulmonary-fibrosis effect from the perspective of inhibiting epithelial cell senescence. The results further enrich the pharmacological application of senegenin and provide novel insights into the pharmacological treatment of idiopathic pulmonary fibrosis.

## 2. Materials and Methods

### 2.1. Animal Experiment

Eight–week–old male *C57BL/6 mice* without underlying diseases were housed in a pathogen–free environment at the animal center of Central South University. After 1 week of acclimatization feeding, the *mice* were anesthetized with sodium pentobarbital. A lung fibrosis model was constructed via intratracheal injection of 50 μL of bleomycin (3 mg/kg; Nippon Kayaku, Tokyo, Japan).

From a total of 110 *mice*, 10 were randomly selected as controls, and the remaining *mice* were used for bleomycin modeling. Control *mice* were injected intraperitoneally with the solvent on days 15–28 after tracheal injection of 50 μL of saline. Meanwhile, on the 14th day after the tracheal injection of bleomycin, the model *mice* were stratified into four groups, each with the same number of *mice* according to their body weights: a lung fibrosis model group, which was injected intraperitoneally with the solvent on days 15–28; a high-dose group, which was injected intraperitoneally with 20 mg/kg of senegenin (Solarbio, Beijing, China) on days 15–28; a medium-dose group, which was injected intraperitoneally with 5 mg/kg of senegenin on days 15–28; and a low-dose group, which was injected intraperitoneally with 1 mg/kg of senegenin on days 15–28. The *mice* were euthanized on day 29, and the lung tissues were obtained.

From a total of 100 *mice*, 10 were randomly selected as controls and the remaining were used for modeling. The control *mice* were treated as described above. On the 14th day after the tracheal injection of bleomycin, the successfully constructed *mouse* models were divided into three groups, with the same number of *mice* according to body weight: a lung fibrosis model group and high-dose group, whose *mice* were treated as described above; and an EX527 group, whose *mice* were pretreated for 30 min with an intraperitoneal injection of 5 mg/kg of EX527 (Selleck Chemicals, Houston, TX, USA) on days 15–28, followed by intraperitoneal injection of 20 mg/kg of senegenin. This study was carried out in accordance with the laboratory animal welfare and ethical principles. The experimental protocol was approved by the Laboratory Animal Welfare and Ethical Committee of Central South University.

We formulated the in vivo injection of senegenin and EX527 according to the solvent formulation recommended by Selleck Chemicals. We first solubilized senegenin or EX527 with DMSO and then added PEG300, Tween80, and saline sequentially, resulting in a final ratio of 5%:40%:5%:50% (*V:V:V:V:V*) for DMSO, PEG300, Tween80, and saline, respectively.

### 2.2. Hematoxylin–Eosin (HE) Staining, Masson Staining, and Ashcroft Score

*Mouse* lung tissue was fixed with 4% paraformaldehyde, embedded in paraffin, and cut into sections. After deparaffinization, the sections were stained with HE (Pinuofei Biological, Wuhan, China) and Masson stain (Pinuofei Biological) and finally dehydrated and sealed. The Ashcroft scoring method based on Ashcroft et al. was used to score the fibrosis level of each HE-stained section, and then the mean value was calculated [21]. Assessment was performed by three researchers.

### 2.3. Determination of Hydroxyproline in Lung Tissue

A homogenate of *mouse* lung tissue was prepared using a hydroxyproline assay kit (Nanjing Jiancheng Biotechnology Institute, Nanjing, China) according to the manufacturer’s instructions and incubated at room temperature. After centrifugation, the absorbance value of the supernatant at 550 nm was measured, and the hydroxyproline level was calculated according to the absorbance value.

### 2.4. Immunofluorescence Staining

Immunofluorescence was performed using a tyramide signal amplification (TSA) Immunofluorescence Kit (Aifang Biological, Changsha, China) according to the manufacturer’s instructions.

For the immunofluorescence staining of lung tissue, the sections were deparaffinized to repair the antigen and block the endogenous peroxidase and the nonspecific antigen. The sections were incubated with anti-P21 antibody (Proteintech, Wuhan, China) overnight. The next day, the sections were incubated with a horseradish-peroxidase-(HRP)-conjugated secondary antibody, followed by incubation with a TSA fluorescent dye. The above steps were repeated, with modifications of anti-SPC antibody (Proteintech) incubation and nuclear staining with DAPI dye. The slides were sealed, and images were captured using a fluorescence microscope.

For cellular immunofluorescence staining, the cells were fixed with 4% paraformaldehyde, blocked for nonspecific antigens after membrane rupture, incubated with anti-P21 antibody overnight, and then incubated with HRP-conjugated secondary antibody. Incubation with TSA fluorescent dye reactive solution was then performed, followed by nuclear staining with DAPI dye. The slides were blocked, and images were captured using a fluorescence microscope.

### 2.5. ROS Staining

For ROS staining of lung tissue, frozen sections of lung tissue were heated to room temperature and were circled using an immunohistochemical pen, after which a fluorescent brightening agent was added. The tissues were incubated with ROS dye, which was followed by nuclear staining with DAPI. The sections were sealed, and images were captured via fluorescence microscopy.

For cellular ROS staining, cells were incubated with ROS fluorescent probe (Beyotime Biotechnology, Shanghai, China) at 37 °C for 30 min and then washed twice with DMEM. Images were then captured via fluorescence microscopy, and ROS levels were measured using flow cytometry.

### 2.6. Determination of Malondialdehyde (MDA) and Glutathione (GSH) Content and Total Superoxide Dismutase (SOD) Activity in Lung Tissue

Total SOD activity and MDA and GSH contents were measured using an MDA, GSH, and SOD assay kit (Nanjing Jiancheng Biotechnology Institute) according to the manufacturer’s instructions and with reference to our previous research method. The *mouse* lung tissue was made into tissue homogenate, and each reagent in the kit was sequentially added. The corresponding absorbance values were measured using each enzyme marker and were calculated according to the instructions.

### 2.7. RNA Extraction and Real-Time Quantitative Polymerase Chain Reaction (qPCR)

RNA was extracted from cells and lung tissues with TRIzol (Thermo Fisher Scientific, Waltham, MA, USA) and reverse transcribed to cDNA using a reverse transcription kit (Thermo Fisher Scientific). The primer sequences (Sangon Biotech, Shanghai, China) are listed in Table 1.

### 2.8. Western Blotting

*Mouse* lung tissues or cells were extracted using radioimmunoprecipitation assay lysate containing phenylmethylsulfonyl fluoride, phosphatase inhibitor, and protease inhibitor (Solarbio), and the total protein concentration was determined using bicinchoninic acid assay kits (CWBIO, Taizhou, China). The proteins were subjected to sodium dodecyl sulfate–polyacrylamide gel electrophoresis and were then transferred to polyvinylidene fluoride membranes. The membranes were incubated with 5% skimmed milk, which was followed by incubation with β-actin, α-SMA, Pgc-1α, and Sirt1 antibodies (Proteintech) and with collagen I, p21, and p16 antibodies (Abcam, Cambridge, UK). The membranes were then incubated with HRP-labeled goat anti-rabbit immunoglobulin G monoclonal antibody (Proteintech) or HRP-labeled goat anti-*mouse* immunoglobulin G monoclonal antibody (Proteintech) at room temperature and washed with Tris-buffered saline with Tween 20 before protein expression was visualized with an ECL developer.

### 2.9. Cell Culture and Hydrogen–Peroxide–Induced Epithelial Cell Senescence

*Mouse* lung epithelial cells (MLE-12 cells) were cultured in a humidified incubator at 37 °C with 5% CO_2_. Induction of cellular senescence was performed as described in several previous studies. The cells were seeded in plates and incubated with medium containing 200 µΜ of hydrogen peroxide (Sigma, St. Louis, MO, USA) for 2 h, which was then changed to normal medium for 48 h.

### 2.10. Cell Viability Assay

Cell viability was determined using Cell Counting Kit-8 (CCK8; Elabscience, Wuhan, China) according to the manufacturer’s instructions. The cells were seeded in 96-well plates and incubated with different concentrations of senegenin for 48 h. The cells were washed with phosphate-buffered saline, treated with CCK-8 reagent, and incubated in an incubator at 37 °C. Finally, the absorbance values at 450 nm were measured.

### 2.11. SiRNA–Specific Gene Silencing

Cells were seeded in well plates and transfected upon reaching 50% confluency. Transfection complexes were prepared by mixing transfection reagents (Rebobio, Guangzhou, China) with SiRNAs (control and Sirt1 SiRNAs from Santa Cruz Biotech (Santa Cruz, CA, USA); Pgc-1α SiRNA from Sangon Biotech). The cells were incubated at room temperature for 15 min and were washed twice with DMEM, and the transfection complexes were then mixed with complete medium without penicillin–streptomycin mixture and added to the well plates before subsequent interventions.

### 2.12. Statistical Analysis

All data were analyzed using Graphpad Prism 8.3.1. All data are expressed as the mean ± standard deviation. Comparisons between two measures were performed using the *t*-test. Comparisons between multiple measures were performed using one-way analysis of variance followed by Tukey’s test. Statistical significance was set at a *p*-value < 0.05.

## 3. Results

### 3.1. Senegenin Inhibited Bleomycin-Induced Pulmonary Fibrosis in Mice

**Figure 1:**
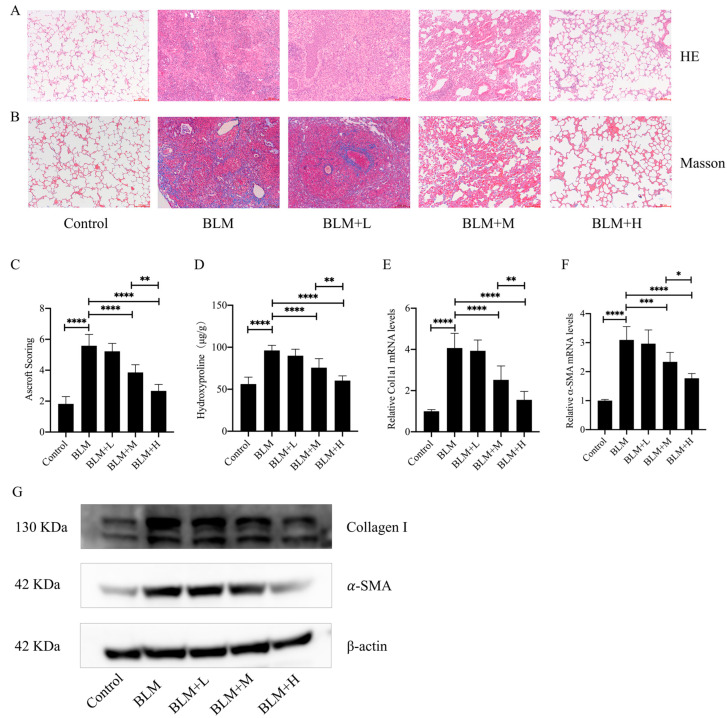
Senegenin improved the pathological changes in lung tissue morphology and structure and reduced the levels of lung fibrosis markers in *mice* with pulmonary fibrosis. High (20 mg/kg), medium (5 mg/kg), and low (1 mg/kg) doses of senegenin were intraperitoneally injected into *mice* on days 15–28 after tracheal injection of bleomycin to examine the therapeutic effects of senegenin on pulmonary fibrosis. (**A**,**B**) HE and Masson staining were used to assess the changes in lung tissue structure and extracellular matrix in *mice*. (**C**) The Ashcroft scoring method was used to comprehensively analyze the degree of lung fibrosis in each group of *mice*. (**D**) Biochemical assay was used to determine the hydroxyproline content in *mouse* lung tissues. (**E**,**F**) qPCR was used to analyze the mRNA expression levels of *ACTA2* and *Col1a1* in lung tissues. (**G**) Western blotting was used to determine the levels of α-SMA and type I collagen in *mouse* lung tissues. Control—control group; BLM—lung fibrosis model group; BLM + L—low-dose treatment group; BLM + M—medium-dose treatment group; BLM + H—high-dose treatment group. (Data are expressed as the mean ± standard deviation; *n* = 8, * *p* < 0.05; ** *p* < 0.01; *** *p* < 0.001; **** *p* < 0.0001).

To investigate the direct antifibrotic effect of senegenin, we constructed a *mouse* lung fibrosis model using bleomycin and administered low, medium, and high doses of senegenin intraperitoneally on days 15–28 after establishment of the model. HE and Masson staining showed that medium and high doses of senegenin effectively improved the lung tissue structure and reduced extracellular matrix deposition in lung tissues of *mice* with lung fibrosis (Figure 1A,B). The Ashcroft score and hydroxyproline determination showed that medium and high doses of senegenin effectively improved the overall pathological changes and collagen deposition in the lungs of *mice* with pulmonary fibrosis (Figure 1C,D). In addition, medium and high doses of senegenin effectively reduced the mRNA and protein expression levels of fibrosis markers type I collagen and α-SMA (Figure 1E–G). These results suggest that medium and high doses of senegenin effectively reduce bleomycin-induced pulmonary fibrosis, with high doses having a more significant effect.

### 3.2. Senegenin Reduced Oxidative Stress Levels in the Lung Tissues of Pulmonary Fibrosis Mice

**Figure 2:**
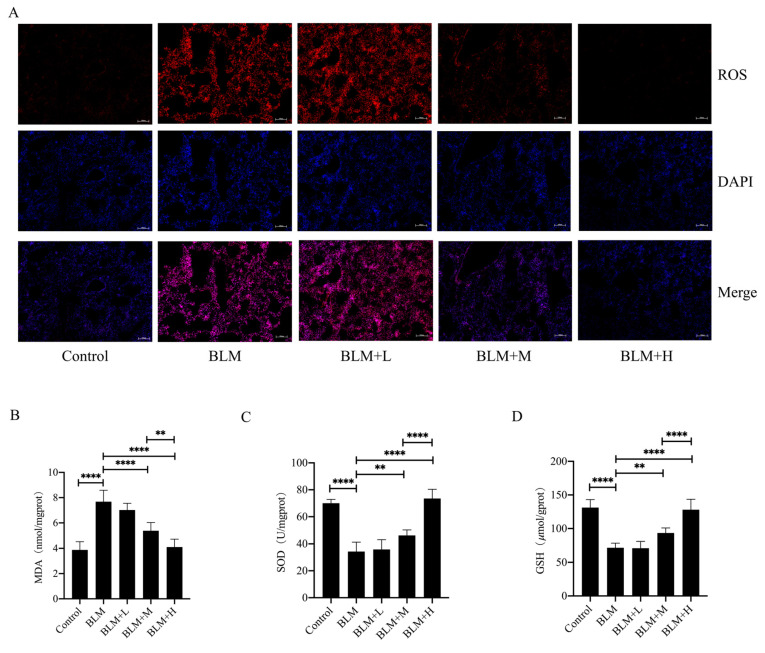
Senegenin reduced ROS and MDA levels and increased GSH levels and total SOD activity in lung tissues of *mice* with pulmonary fibrosis. (**A**) DHE staining was used to detect ROS (red) levels in *mouse* lung tissues. (**B**–**D**) Biochemical assays were used to detect MDA and GSH levels and SOD activity. Control—control group; BLM—lung fibrosis model group; BLM + L—low-dose treatment group; BLM + M—medium-dose treatment group; BLM + H—high-dose treatment group. (Data are expressed as the mean ± standard deviation; *n* = 8, ** *p* < 0.01; **** *p* < 0.0001).

Senegenin has been shown to have antioxidative effects [22]. To investigate whether it exerts an antioxidative effect in addition to its antifibrotic effect, we examined the levels of ROS in the lung tissues of different groups of *mice* and found that medium and high doses of senegenin significantly reduced the levels of ROS in the lung tissues of *mice* with pulmonary fibrosis (Figure 2A). Meanwhile, high and medium doses of senegenin effectively reduced the levels of MDA and increased the levels of GSH and total SOD activity in the lung tissues of *mice* with pulmonary fibrosis (Figure 2B–D), with the high dose exhibiting a more pronounced effect. This suggests that senegenin also exerts antioxidative effects along with antifibrotic effects, and its anti–pulmonary-fibrosis effects may also be related to their antioxidative effects.

### 3.3. Senegenin Inhibited Epithelial Cell Senescence in Lung Tissues of Pulmonary Fibrosis Mice

**Figure 3:**
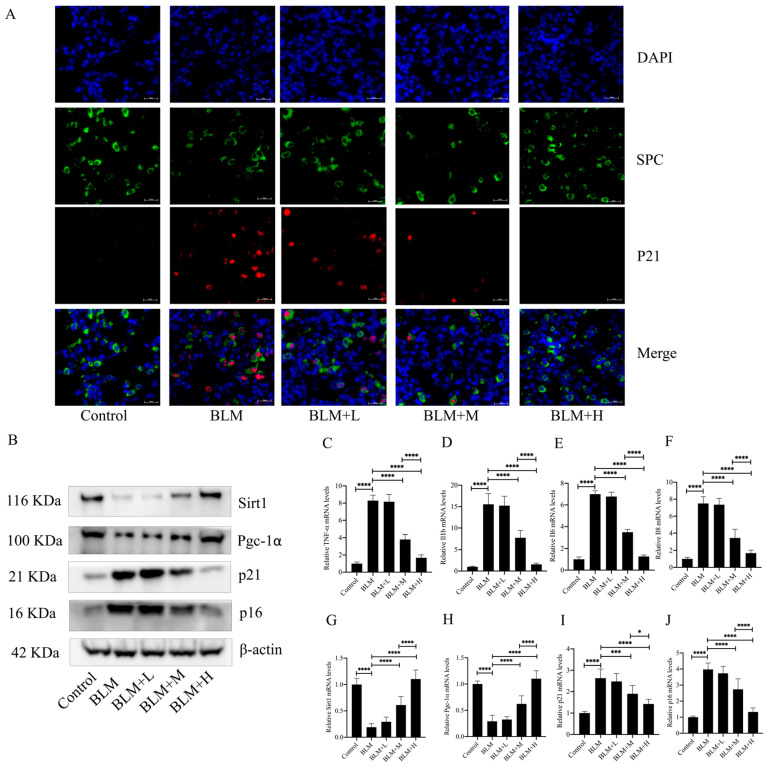
Senegenin reduced epithelial cell senescence; decreased the expression levels of TNF-α, IL1-β, IL-6, IL-8, p21, and p16; and increased the expression levels of Sirt1 and Pgc-1α in lung tissues of *mice* with pulmonary fibrosis. (**A**) Immunofluorescence staining of SPC (green), P21 (red) and DAPI (blue) was used to detect the number of senescent epithelial cells in *mouse* lung tissues. (**B**) Western blotting was used to detect the protein expression levels of Sirt1, Pgc-1α, p21, and p16 in *mouse* lung tissues. (**C**–**J**) qPCR was used to detect the mRNA expression levels of *TNF-α*, *IL1-β*, *IL-6*, *IL-8*, *p21*, *p16*, *Sirt1*, and *Pgc-1α* in *mouse* lung tissues. Control—control group; BLM—lung fibrosis model group; BLM + L—low-dose treatment group; BLM + M—medium-dose treatment group; BLM + H—high-dose treatment group. (Data are expressed as the mean ± standard deviation; *n* = 8, * *p* < 0.05; *** *p* < 0.001; **** *p* < 0.0001).

Although the specific pathogenesis of IPF is currently unknown, epithelial cell senescence has been shown to be an important pathological change in the development of the disease. Oxidative stress is one of the important factors leading to epithelial senescence, whereas inhibition of epithelial cell senescence inhibits the progression of IPF [23]. In the results described above, we found that senegenin exerted its antifibrotic effect along with its antioxidative effect. We then investigated whether the antifibrotic effect of senegenin is related to its antisenescent effect on epithelial cells. The results showed that medium and high doses of senegenin significantly reduced epithelial cell senescence (Figure 3A) and the levels of four senescence-associated secretory phenotype (SASP) secretions, namely *TNF-α*, *IL1-β*, *IL-6*, and *IL-8*, in lung tissues of mice with pulmonary fibrosis (Figure 3C–F). Medium and high doses of senegenin also significantly reduced the protein and mRNA expression levels of the senescence markers p21 and p16 (Figure 3B,I–J). Sirt1 plays a key role in exerting antiaging effects in the body, and activation of the Sirt1/Pgc-1α pathway has been demonstrated to be an important pathway by which Sirt1 exerts its antiaging effects [24]. To investigate whether the mechanism of the antisenescence of epithelial cells exerted by senegenin is related to the activation of the Sirt1/Pgc-1α pathway, we examined the expression levels of Sirt1 and Pgc-1α in *mouse* lung tissues. The results showed that medium and high doses of senegenin significantly increased the protein and mRNA expression levels of Sirt1 and Pgc-1α in the lung tissues of pulmonary fibrosis *mice* (Figure 3B,G–H). The above results indicated that senegenin exerts an antisenescent effect, which might be related to its activation of the Sirt1/Pgc-1α pathway, and that this effect might also be related to its antifibrotic effect.

### 3.4. Senegenin Inhibited Hydrogen-Peroxide-Induced Epithelial Cell Senescence

**Figure 4:**
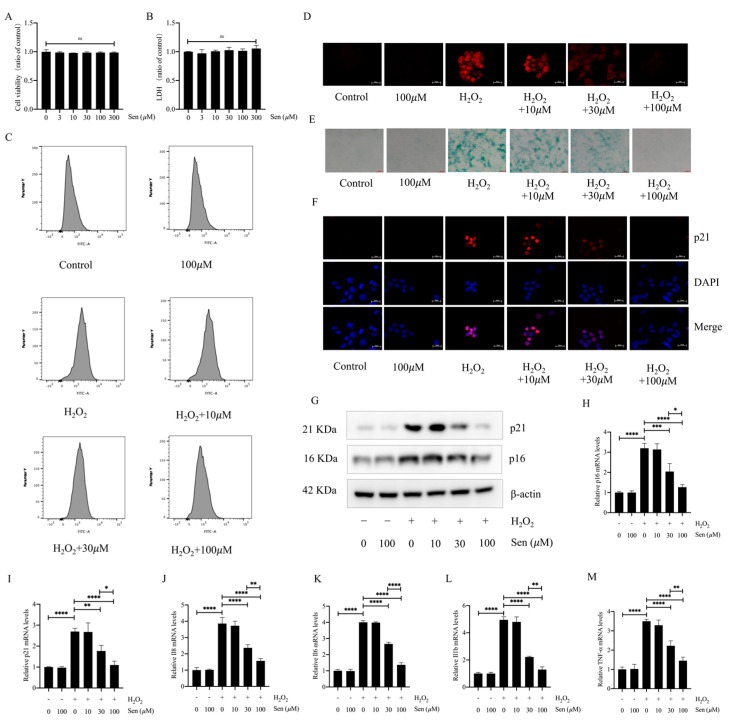
Senegenin decreased the levels of ROS and β-galactosidase in epithelial cells and reduced the expression levels of TNF-α, IL1-β, IL-6, IL-8, p21, and p16 in lung tissues of *mice* with pulmonary fibrosis. (**A**) Cell viability was detected using CCK8 assay. (**B**) Biochemical assays were used to detect cell supernatant LDH content. (**C**) Flow cytometry was used to detect ROS content in epithelial cells incubated with ROS probes. (**D**) Fluorescence microscopy was used to detect ROS (red) content in epithelial cells incubated with ROS probes. (**E**) β–galactosidase staining was performed to detect the expression level of β-galactosidase. (**F**) Immunofluorescence was used to detect the expression level of cellular p21 (red) and DAPI (blue). (**G**) Western blotting was used to detect the protein expression levels of p21 and p16. (**H**,**I**) qPCR was used to detect the mRNA expression levels of *p21* and *p16*. (**J**–**M**) qPCR was used to detect the mRNA expression levels of *TNF*-*α*, *IL1*-*β*, *IL*-*6*, and *IL*-*8*. (Data are expressed as the mean ± standard deviation, and all experiments were repeated independently at least thrice; * *p* < 0.05; ** *p* < 0.01; *** *p* < 0.001; **** *p* < 0.0001).

To further investigate whether the antifibrotic effect of senegenin is related to its antisenescent effect, we explored the effect of senegenin on hydrogen-peroxide-induced epithelial cell senescence. We first examined the effect of different concentrations of senegenin on the viability and supernatant lactate dehydrogenase (LDH) content of lung epithelial cells to determine the concentration to be used, and the results showed that concentrations of 0–300 μΜ did not significantly affect epithelial cell viability (Figure 4A,B). Oxidative stress is one of the main factors leading to cellular senescence, and the level of ROS increases significantly during cellular senescence [25]. In the results described above, we found that senegenin significantly reduced the level of oxidative stress in lung tissues of *mice* with pulmonary fibrosis. We similarly found that 30 µΜ versus 100 µΜ of senegenin significantly reduced the level of ROS during hydrogen-peroxide-induced senescence of epithelial cells (Figure 4C,D). In addition, staining of cellular senescence-associated β-galactosidase and measurement of the expression levels of p21, p16, *TNF-α*, *IL1-β*, *IL-6*, and *IL-8* revealed that 30 µΜ versus 100 µΜ of senegenin significantly reduced the expression levels of these indicators (Figure 4E–M). Thus, senegenin significantly inhibits hydrogen-peroxide-induced epithelial cell senescence, which may also be the cellular mechanism for its anti-pulmonary-fibrosis effect.

### 3.5. The Sirt1/Pgc-1α Pathway Mediated the Antisenescent Effect of Senegenin

**Figure 5:**
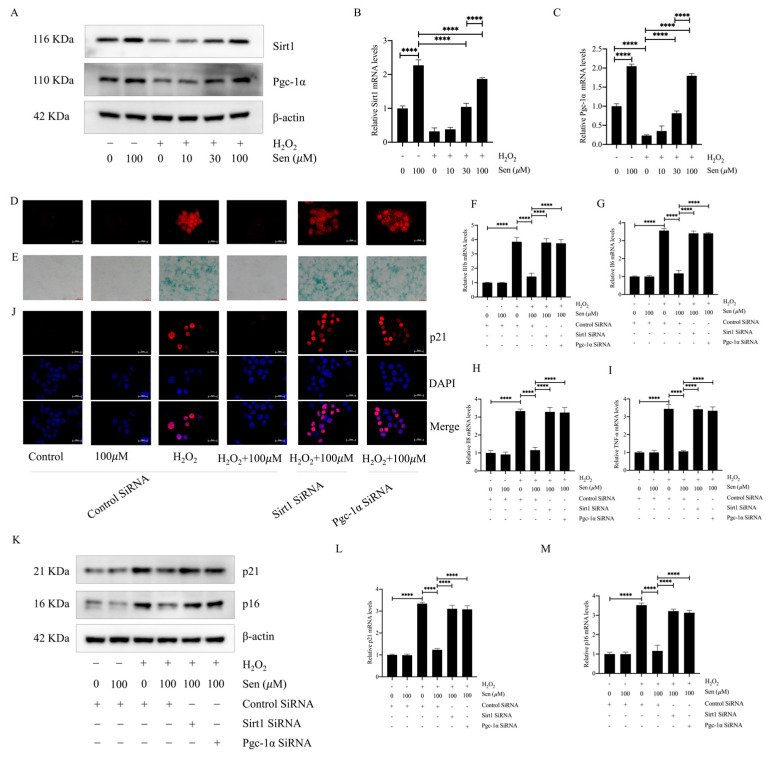
Senegenin increased the expression levels of Sirt1 and Pgc-1α, and silencing of each gene inhibited the effects of senegenin on ROS, β-galactosidase, p21, p16, TNF-alpha, IL1-β, IL-6, and IL-8. (**A**) Western blotting was used to detect the protein expression levels of Sirt1 and Pgc-1α. (**B**,**C**) qPCR was used to detect the mRNA expression levels of *Sirt1* and *Pgc-1α*. (**D**) Fluorescence microscopy was used to detect ROS (red) levels in epithelial cells incubated with ROS probes. (**E**) Biochemical assay was used to detect β-galactosidase content in cells. (**F**–**I**) qPCR was used to detect the mRNA expression levels of *TNF*-*α*, *IL1*-*β*, *IL*-*6*, and *IL*-*8*. (**J**) Immunofluorescence was used to detect the expression level of p21 (red) and DAPI (blue). (**K**) Western blotting was used to detect the protein expression levels of p21 and p16. (**L**,**M**) qPCR was used to detect the mRNA expression levels of *p21* and *p16*. (Data are expressed as the mean ± standard deviation, and all experiments were repeated independently at least thrice; **** *p* < 0.0001).

To further investigate the mechanism of antisenescence of epithelial cells by senegenin, we examined its effects on Sirt1 and Pgc-1α, considering that senegenin significantly increased Sirt1 and Pgc-1α expression in lung tissues of pulmonary fibrosis *mice* and that the Sirt1/Pgc-1α pathway plays an important role in antiaging. The results showed that 30 and 100 µΜ of senegenin significantly increased the protein and mRNA expression levels of Sirt1 and Pgc-1α (Figure 5A–C). Silencing of the *Sirt1* and *Pgc*–*1α* genes with siRNA resulted in the inhibition of the effects of senegenin on the ROS and β-galactosidase content and p16, p21, *TNF*–*α*, *IL1*–*β*, *IL*–*6*, and *IL*–*8* expression levels during hydrogen-peroxide-induced epithelial cellular senescence (Figure 5D–M). Thus, the inhibitory effect of senegenin on epithelial cell senescence was mediated via the Sirt1/Pgc-1α pathway.

### 3.6. Sirt1 Mediated the In Vivo Antifibrotic Effects of Senegenin

**Figure 6:**
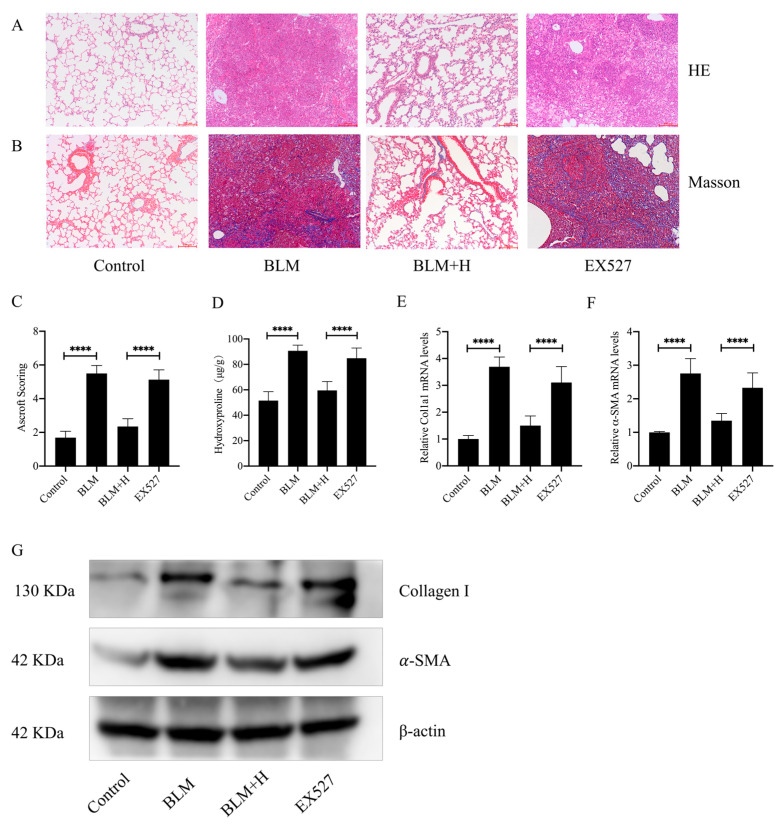
Sirt1 inhibitor EX527 inhibited the effect of senegenin on lung-fibrosis-related indices. EX527 (5 mg/kg) was intraperitoneally injected 30 min prior to high-dose senegenin treatment in pulmonary fibrosis *mice* modeled using bleomycin, and the results were compared with those in high–dose senegenin–treated pulmonary fibrosis *mice*. (**A**,**B**) HE and Masson staining were used to assess the changes in lung tissue structure and extracellular matrix in *mice*. (**C**) The Ashcroft score was used to comprehensively analyze the degree of lung fibrosis in each group of *mice*. (**D**) Biochemical assay was used to detect the hydroxyproline content in *mouse* lung tissues. (**E**,**F**) qPCR was used to analyze the mRNA expression levels of *ACTA2* and *Col1a1* in lung tissues. (**G**) Western blotting was used to detect the levels of α-SMA and type I collagen in *mouse* lung tissues. Control—control group; BLM—lung fibrosis model group; BLM + H—high-dose treatment group; EX527—group treated with EX527 before high-dose treatment. (Data are expressed as the mean ± standard deviation; *n* = 8; **** *p* < 0.0001).

To further validate the role of Sirt1 in the anti–pulmonary fibrosis activity of senegenin in vivo, we observed the effects of senegenin on pulmonary fibrosis *mice* administered with a Sirt1-specific inhibitor EX527 and subsequently treated with a high dose of senegenin. The results showed that EX527 inhibited the effects of senegenin on the disordered lung tissue outcome with extracellular matrix overdeposition in pulmonary fibrosis *mice* (Figure 6A–D). EX527 also inhibited the effects of senegenin on the reduction of α-SMA and type I collagen levels in the lung tissue of pulmonary fibrosis *mice* (Figure 6E–G). These results indicate that EX527 significantly inhibits the anti-pulmonary-fibrosis effects of senegenin, suggesting that the in vivo antifibrotic effects of senegenin are mediated by Sirt1.

### 3.7. Sirt1 Mediated the In Vivo Antioxidative Effect of Senegenin

**Figure 7:**
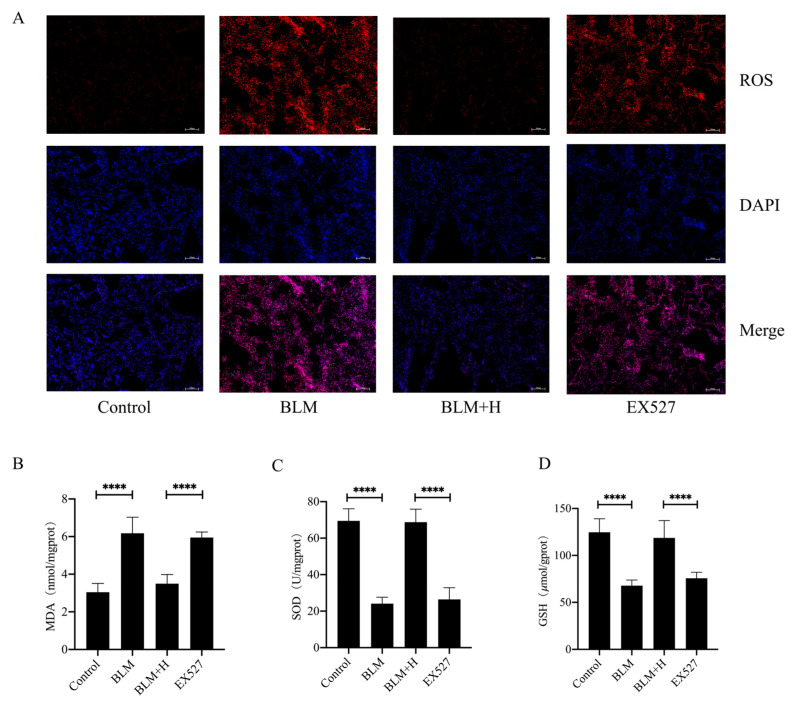
EX527 inhibited the effects of senegenin on ROS, MDA, and GSH levels and SOD activity in lung tissues of pulmonary fibrosis *mice*. (**A**) DHE staining was used to detect ROS (red) levels in the lung tissues of *mice* in each group. (**B**–**D**) Biochemical assays were used to detect MDA and GSH levels and SOD activity. Control—control group; BLM—lung fibrosis model group; BLM + H—high-dose treatment group; EX527—group treated with EX527 before high-dose treatment. (Data are expressed as the mean ± standard deviation; *n* = 8; **** *p* < 0.0001).

To investigate whether Sirt1 also mediated the in vivo antioxidative effects of senegenin, *mouse* lung tissues were stained for ROS, and we found that EX527 inhibited the ROS-lowering effect of senegenin in lung tissues of pulmonary fibrosis *mice* (Figure 7A). Similarly, EX527 also inhibited the reduction of MDA and the elevation of GSH levels and total SOD activity by senegenin in the lung tissues of pulmonary fibrosis *mice* (Figure 7B–D). These results suggest that Sirt1 also mediates the in vivo antioxidative effects of senegenin.

### 3.8. Sirt1 Mediated the Antisenescent Effect of Senegenin In Vivo

**Figure 8:**
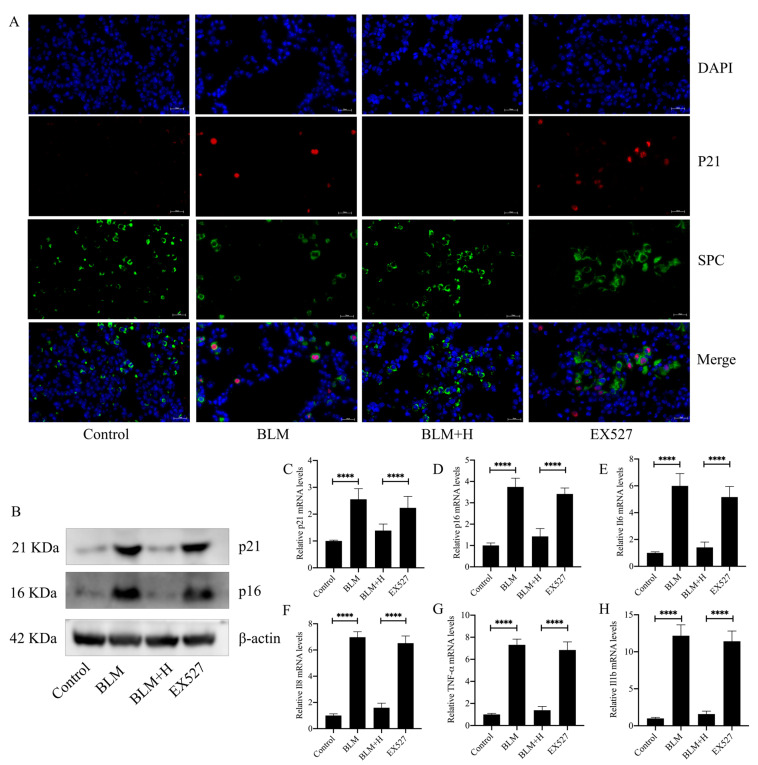
EX527 inhibited the effect of senegenin on epithelial cell senescence and on the expression of p21, p16, TNF-α, IL1-β, IL-6, and IL-8 in lung tissues of pulmonary fibrosis *mice*. (**A**) Immunofluorescence staining of SPC (green), P21 (red) and DAPI (blue) was used to detect the number of senescent epithelial cells in *mouse* lung tissues. (**B**) Western blotting was used to detect the protein expression levels of p21 and p16 in *mouse* lung tissues. (**C**–**H**) qPCR was used to detect the expression levels of *p21*, *p16*, *TNF-α*, *IL1-β*, *IL-6*, and *IL-8* in *mouse* lung tissues. Control—control group; BLM—lung fibrosis model group; BLM + H—high-dose treatment group; EX527—group treated with EX527 before high-dose treatment. (Data are expressed as the mean ± standard deviation; *n* = 8, **** *p* < 0.0001).

To further characterize the Sirt1-mediated antifibrotic effects in relation to its antisenescent effects on epithelial cells, we examined the effect of EX527 on indicators related to epithelial cell senescence. The results showed that EX527 inhibited the role of senegenin in inhibiting epithelial cell senescence (Figure 8A). Similarly, the inhibitory effects of senegenin on the expression of p21 and p16 in lung tissues of pulmonary fibrosis *mice*, along with the inhibitory effects of secretion of various SASPs, were inhibited by EX527 (Figure 8B–H). These results demonstrate that Sirt1 mediates the antisenescent effect of senegenin on epithelial cells and further shows that the antisenescent effect of senegenin mediates its antifibrotic effect.

## 4. Discussion

In the present study, we demonstrated, for the first time, that senegenin inhibited alveolar epithelial cell senescence induced by oxidative stress through the activation of the Sirt1/Pgc-1α pathway, thereby exerting an antifibrotic effect on lungs. Activation of the Sirt1/Pgc-1α signaling pathway has been shown to exert antifibrotic effects through effects on the FOXO3 pathway, the PI3K/AKT pathway, and the Nrf2 pathway [26,27,28], and thus these pathways may also be relevant to the antifibrotic effects exerted by senegenin. In addition, activation of the Sirt1/Pgc-1α signaling pathway has been shown to exert anti-oxidative stress effect, ameliorate mitochondrial dysfunction, and inhibit apoptosis, which are all closely related to the development of pulmonary fibrosis [24,29,30]. Thus, the ability of senegenin to exert antifibrotic effects on lung fibrosis through the activation of the Sirt1/Pgc-1α signaling pathway may also be related to its modulation of oxidative stress, mitochondrial dysfunction, and apoptosis. All of the above deserve to be further explored in subsequent studies.

Oxidative stress has an important role in cellular senescence and the development of IPF [23,31,32,33]. For one, ROS may drive cellular senescence through DNA damage and the activation of DNA-damage-response-associated pathways, and the senescence of different cells, including epithelial cells, leads to the progression of idiopathic pulmonary fibrosis. For another, mitochondrial dysfunction occurring in senescent cells leads to more ROS production, which may further contribute to the progression of cellular senescence and fibrosis [34]. We investigated the anti-pulmonary-fibrosis effects of senegenin and the underlying molecular mechanisms using a *mouse* lung fibrosis model and a stress-induced alveolar epithelial premature senescence model, which were constructed using bleomycin and hydrogen peroxide, respectively, and the results showed that senegenin exerted antioxidative and antisenescent effects both in vivo and in vitro. Considering the important role of oxidative stress in alveolar epithelial cell senescence and in idiopathic pulmonary fibrosis, we postulate that the anti-pulmonary-fibrosis effect of senegenin may be closely related to its inhibition of oxidative-stress-induced epithelial cell senescence. Studies have confirmed that the antioxidative effect of senegenin is involved in their neuroprotective effects in the treatment of Parkinson’s disease [35], and whether the therapeutic effects of senegenin on Parkinson’s and other disorders are also related to their anticellular senescence effects warrants further exploration.

Sirtuin proteins are a class of proteins with nicotinamide adenine dinucleotide (NAD+)-dependent deacetylase activity or ADP-ribosyltransferase activity, and seven Sirtuin proteins (from Sirt1 to Sirt7) have been identified in mammals, of which Sirt1 is the most extensively studied [36]. Sirtuin proteins have a wide range of functions, among which the role of Sirt1 in antiaging has been widely validated [37]. Owing to its antiaging effects, Sirt1 is used as a key target for the treatment of a variety of diseases, including idiopathic pulmonary fibrosis, tumors, osteoporosis, and atherosclerosis [38,39,40,41]. In the present study, we found that senegenin significantly increased the expression level of Sirt1 in the lung tissues of pulmonary fibrosis *mice* and alveolar epithelial cells in vivo and in vitro, respectively. Moreover, the antifibrotic and antisenescent effects of senegenin were suppressed in vivo by treatment with the Sirt1 inhibitor EX527, and the antioxidative and antisenescent effects of senegenin were suppressed in vitro by silencing the Sirt1 gene using siRNA, which suggest that Sirt1 is a key target for senegenin in exerting antifibrotic and antisenescent effects. In addition to Sirt1, other proteins of the Sirtuin family such as Sirt3 and Sirt6 play important roles in antiaging and anti-pulmonary fibrosis [16,42]. Whether senegenin, while exerting a role in promoting Sirt1 expression, also affects the expression of other Sirtuin proteins warrants further exploration.

Pgc-1α is a key mediator of mitochondrial energy metabolism and has been shown to be a key regulator of cellular responses to a variety of physiological stimuli [43,44,45]. The Sirt1/Pgc-1α axis exerts a positive effect in various diseases by inhibiting cellular senescence [46,47,48]. However, the role of Pgc-1α in pulmonary fibrosis is currently unknown. When we explored the effects of senegenin on Sirt1 in vivo and in vitro, we similarly found that senegenin promoted the expression of Pgc-1α and that the senegenin-induced antioxidative and antisenescent effects on alveolar epithelial cell were suppressed upon specific silencing of Pgc-1α. This suggests that the Sirt1/Pgc-1α pathway mediates the antioxidative and antisenescent effects of senegenin. In addition, Pgc-1α is closely related to the function of the mitochondria, which play an important role in cellular senescence; however, we did not test the effects of senegenin on mitochondrial function in this study. Our subsequent studies should examine the effects of Sirt1/Pgc-1α on the mitochondrial function of epithelial cells.

In addition to antioxidative effects, senegenin has also been reported to have antiapoptotic, anti–inflammatory, and anti-injury pharmacological effects [49,50,51], and whether these pharmacological effects are related to its antioxidative and antisenescent effects warrants further exploration. The development of pulmonary fibrosis involves a variety of pathological mechanisms, which also include apoptosis, inflammation, cellular senescence, and oxidative stress injury. In our study, we demonstrated that senegenin exerts its anti–pulmonary fibrosis effect through its anti-cellular senescence effect, and whether the anti–pulmonary fibrosis effect of senegenin is also related to the other pharmacological effects mentioned above deserves to be further explored. The present study showed that senegenin inhibits oxidative-stress-induced epithelial cell senescence through the Sirt1/Pgc-1α pathway, thus exerting an antifibrotic effect on lung fibrosis, but whether senegenin has synergistic or other effects with drugs commonly used for the treatment of IPF, including pirfenidone and nintedanib, were not explored in this study. It is important to explore these interactions, which will provide a better scientific basis for the clinical application of senegenin in the future, and we will further explore the relevant elements in future studies. This study is the first to propose the Sirt1/Pgc-1α pathway as a novel drug target for the treatment of pulmonary fibrosis by inhibiting alveolar epithelial cell senescence. This proposition has important theoretical value, as it reveals a novel molecular mechanism for the treatment of pulmonary fibrosis. Furthermore, from a clinical perspective, targeting the Sirt1/Pgc-1α pathway may enable the development of innovative therapeutic strategies for the treatment of pulmonary fibrosis, which could help address the critical medical need for pulmonary fibrosis therapy.

## Figures and Tables

**Table 1:** The primer sequence.

Gene Name	Forward [5′–3′]	Reverse [5′–3′]
*Mouse collagen I*	GAGCGGAGAGTACTGGATCG	GCTTCTTTTCCTTGGGGTTC
*Mouse α-SMA*	TGGCTATTCAGGCTGTGCTGTC	CAATCTCACGCTCGGCAGTAGT
*Mouse β-Actin*	GTGCTATGTTGCTCTAGACTTCG	ATGCCACAGGATTCCATACC
*Mouse p21*	CCTGGTGATGTCCGACCTGTTC	CGAAGTCAAAGTTCCACCGTTCTC
*Mouse p16*	AAGAGCGGGGACATCAAGACATC	AAAGACCACCCAGCGGAACAC
*Mouse * *Pgc-1* *α*	TTCGCTGCTCTTGAGAATGGATATAC	TCGTCTGAGTTGGTATCTAGGTCTG
*Mouse Sirt1*	GTGGCAGTAACAGTGACAGTGG	TCCAGATCCTCCAGCACATTCG
*Mouse TNF-α*	GTGCCTATGTCTCAGCCTCTTCTC	TGGTTTGTGAGTGTGAGGGTCTG
*Mouse IL-1β*	CTCGCAGCAGCACATCAACAAG	CCACGGGAAAGACACAGGTAGC
*Mouse IL-6*	TTCTTGGGACTGATGCTGGTGAC	GTGGTATCCTCTGTGAAGTCTCCTC
*Mouse IL-8*	CTCCTGCTGGCTGTCCTTAACC	TGGGACTGCTATCACTTCCTTTCTG

## Data Availability

Any necessary data from this study can be obtained by contacting the corresponding authors.
